# A Comprehensive Primer on Radiation Oncology for Non-Radiation Oncologists

**DOI:** 10.3390/cancers15204906

**Published:** 2023-10-10

**Authors:** Arnaud Beddok, Ruth Lim, Juliette Thariat, Helen A. Shih, Georges El Fakhri

**Affiliations:** 1 Department of Radiation Oncology, Institut Godinot, 51100 Reims, France; 2 Gordon Center for Medical Imaging, Massachusetts General Hospital, Harvard Medical School, Boston, MA 02114, USA; 3 Department of Radiology, Massachusetts General Hospital, Harvard Medical School, Boston, MA 02114, USA; 4 Department of Radiation Oncology, Centre François-Baclesse, 14000 Caen, France; 5 Department of Radiation Oncology, Massachusetts General Hospital, Harvard Medical School, Boston, MA 02114, USA

**Keywords:** multidisciplinary teams, radiation oncology, non-oncology physicians, personalized medicine

## Abstract

**Simple Summary:**

The gap in understanding of radiation therapy (RT) procedures among non-radiation oncologists poses a significant barrier to optimal cancer care. Aimed at equipping non-oncologists, medical students, and non-clinical researchers with crucial insights into RT, this paper serves as a comprehensive guide that demystifies the intricate, multi-step journey from treatment planning to patient follow-up. Highlighting the indispensable role of radiation oncologists (ROs) in this process, the paper delves into pivotal phases such as CT simulation, treatment target and organs at risk (OAR) delineation, and acute toxicity management. Ultimately, the manuscript aims to significantly elevate patient care standards by bridging the knowledge gap in non-oncology healthcare circles about RT. Its significance lies in its potential to foster improved interdisciplinary collaboration, thereby enhancing the effectiveness and safety of cancer treatment regimens.

**Abstract:**

**Background:** Multidisciplinary management is crucial in cancer diagnosis and treatment. Multidisciplinary teams include specialists in surgery, medical therapies, and radiation therapy (RT), each playing unique roles in oncology care. One significant aspect is RT, guided by radiation oncologists (ROs). This paper serves as a detailed primer for non-oncologists, medical students, or non-clinical investigators, educating them on contemporary RT practices. **Methods:** This report follows the process of RT planning and execution. Starting from the decision-making in multidisciplinary teams to the completion of RT and subsequent patient follow-up, it aims to offer non-oncologists an understanding of the RO’s work in a comprehensive manner. **Results:** The first step in RT is a planning session that includes obtaining a CT scan of the area to be treated, known as the CT simulation. The patients are imaged in the exact position in which they will receive treatment. The second step, which is the primary source of uncertainty, involves the delineation of treatment targets and organs at risk (OAR). The objective is to ensure precise irradiation of the target volume while sparing the OARs as much as possible. Various radiation modalities, such as external beam therapy with electrons, photons, or particles (including protons and carbon ions), as well as brachytherapy, are utilized. Within these modalities, several techniques, such as three-dimensional conformal RT, intensity-modulated RT, volumetric modulated arc therapy, scattering beam proton therapy, and intensity-modulated proton therapy, are employed to achieve optimal treatment outcomes. The RT plan development is an iterative process involving medical physicists, dosimetrists, and ROs. The complexity and time required vary, ranging from an hour to a week. Once approved, RT begins, with image-guided RT being standard practice for patient alignment. The RO manages acute toxicities during treatment and prepares a summary upon completion. There is a considerable variance in practices, with some ROs offering lifelong follow-up and managing potential late effects of treatment. **Conclusions:** Comprehension of RT clinical effects by non-oncologists providers significantly elevates long-term patient care quality. Hence, educating non-oncologists enhances care for RT patients, underlining this report’s importance.

## 1. Introduction

Despite major innovations in screening, diagnosis, and treatment over the past 20 years, cancer remains a frequent disease and a major cause of mortality worldwide in 2022. In a recent study, Siegel et al., estimated that the number of new cases of invasive cancers in the United States in 2022 will be approximately 1,918,030 cases, equivalent to about 5250 new cases each day [1]. The most common cancers remain breast cancer for women and prostate cancer for men. In addition, cancer remains a high cause of mortality, with an estimated 609,360 cancer deaths in the United States in 2022, which is equivalent to nearly 1700 deaths per day. The largest number of deaths are from lung, prostate, and colorectal cancers for men and lung, breast, and colorectal cancers for women. Among the various cancer treatment modalities, radiation therapy (RT) is one of the most widely used therapies, particularly for early breast cancer, prostate adenocarcinoma, head and neck cancer (HNC), and lung cancer [2]. The whole spectrum of potential therapeutic intentions for RT is broad and evolving, encompassing not only curative and definitive (“exclusive”) approaches but also neoadjuvant and adjuvant settings. In the neoadjuvant context, RT is deployed to shrink tumors before surgical intervention, while adjuvant RT is used postoperatively to minimize the risk of cancer recurrence by eradicating residual microscopic disease [3]. Emerging techniques, such as stereotactic RT, serve as a form of local ablative therapy, delivering high doses of radiation with unparalleled precision to localized tumor sites. This method is increasingly utilized for early-stage cancers and isolated metastatic lesions. Furthermore, RT also finds applications in palliative and supportive care, often being used to alleviate symptoms in cases of advanced or metastatic diseases [2]. Therefore, it is common for a physician, whatever their specialty, to be faced with a patient who is undergoing or has undergone RT. Moreover, the patient’s primary care physician and other health care providers routinely get a summary of the delivered RT, which is essential to integrating long-term follow up care of patients and monitoring of radiation-induced late toxicity [4]. However, RT is actually often poorly understood by non-oncologist physicians. In a recent study that assessed training and understanding of RT among non-oncologist physicians, Siau et al. reported that half of the physicians surveyed believed they were insufficiently trained on the indications for RT, its benefits, and radiation-induced toxicity [5]. Several studies have also shown that non-oncologist physicians’ lack of knowledge about RT was actually a barrier to patients’ access to RT [6]. Conversely, other studies have shown that close collaboration between primary care physicians and radiation oncologists (RO) leads to better patient management, especially for patients with metastatic disease [7]. The aim of the present study was to provide a practical summary on the current most common RT practices for non-oncologists to enhance multidisciplinary management of patients during and after RT.

## 2. Fundamental Aspects

### 2.1. Basics of Radiobiology—Radiation Effects on DNA, Fractionation

Although many targets have been identified in the tumor microenvironment, the primary target of ionizing radiation actually remains the DNA molecule. Radiation-induced cell damage usually results from direct or indirect action of ionizing radiation on DNA [8]. In direct action, the radiation directly impacts the DNA molecule, disrupting the molecular structure. This structural change results in cell damage and even cell death. The damaged cells that survive may subsequently induce carcinogenesis or other abnormalities [9]. This process becomes predominant with high radiation doses [10]. In indirect action, radiation strikes water molecules, the main constituent of the cell, and other organic molecules in the cell, producing free radicals such as hydroxyl (HO•). Free radicals are highly reactive and therefore react with DNA molecules to induce molecular structural damage. The consequence of the indirect action of radiation on DNA molecules is the alteration of functions or cell death. The number of free radicals produced by ionizing radiation is related to the total dose. The majority of radiation-induced damage is the result of the indirect action mechanism since water constitutes nearly 70% of the cell’s composition. Several types of DNA damage have been reported: (1) Single-strand breaks, (2) double-strand breaks (DSB), (3) base damage, (4) sugar damage, (5) DNA cross-linking, and (6) clustered damage sites. The most deleterious lesion and the most serious threat to cells are DSBs. DSBs that occur without effective repair or with a tendency for error can cause carcinogenesis or cell death.

One of the questions most often heard from patients and non-oncologists is about the differential effect of ionizing radiation between tumors and healthy tissue. Actually, DNA damage repair mechanisms are less efficient in tumor cells than in non-tumor cells. Normal cells are able to repair a relatively large amount of the damage caused by radiation, unlike tumor cells, which accumulate this damage, become unable to divide, and eventually die [11]. Mitotic cell death occurs when cells die because they are no longer able to divide [12]. Since the introduction of RT, treatments are divided into fractions to provide time for the normal cells to repair themselves and to avoid serious side effects. In general, conventional fractionation involves delivering 1.8–2.1 Gy per fraction across approximately 10–40 sessions, depending on the specific indications. One of the negative mechanisms of fractionation is tumor repopulation [13]. The molecular mechanisms explaining this phenomenon are not yet fully understood, but if the total time between the first and last treatment sessions is too long, the tumor cells tend to proliferate, and the effectiveness of the treatment may be reduced. These few notions bring us to an essential point of RT: the benefit/risk balance: the benefit of treating the tumor and possibly prolonging the patient’s survival versus the risk of inducing important side effects and altering the quality of life.

### 2.2. Basics of Medical Physics—Radiation Modalities

**Figure 1:**
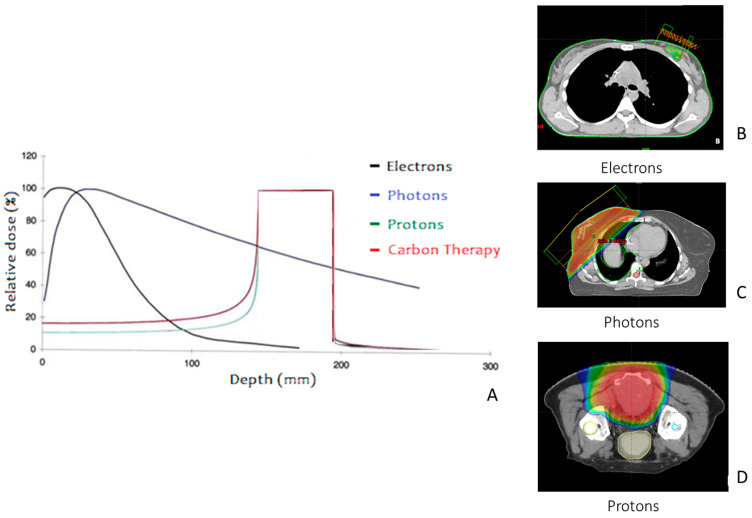
Comparative Dose Deposition of Various Particles in Radiation Therapy. This comprehensive figure visually elucidates the dose deposition profiles of various particles commonly employed in radiation therapy as they penetrate tissue (**A**): Electrons are represented in black, photons in dark blue, protons in green, and carbon ions in red. Adjacent to this, a triptych of images illustrates real-world examples of treatment planning utilizing electrons (**B**), photons (**C**), and protons (**D**). The color gradient signifies the radiation dose levels: warmer hues such as orange indicate high doses, while cooler shades such as blue denote lower doses.

There are currently four types of particles used in clinical RT: photons (most commonly used), electrons (often used), protons, and heavy ions (rarer) [14]. As illustrated in Figure 1, the in-depth dose distribution (Figure 1A) of these particles differs significantly when they enter the tissue. Electrons (Figure 1B) release their entire dose in the first few centimeters of the tissue, making them suitable for treating superficial lesions [15]. In contrast, photons (Figure 1C) deposit their dose more deeply, making them more useful for treating deeper lesions.

Access to protons and carbons (Figure 1D) remains limited due to the scarcity of proton therapy centers; however, protons are highly beneficial for treating lesions in close proximity to critical organs. Indeed, in a uniform medium, monoenergetic protons travel a well-defined distance, losing energy at an increasing rate before coming to a halt [16]. This forms the characteristic Bragg peak (Figure 1). In addition to their unique physical properties, protons and heavy ions have distinct biological advantages, such as a higher linear energy transfer (LET) and a relative higher relative biological effectiveness (RBE) compared to photons [17].

LET quantifies the energy deposition by a charged particle as it moves through a medium, typically given in units of keV/µm. Essentially, it captures the average energy loss by a charged particle for each unit length of its journey. While high-LET radiation such as carbon ions deposits greater amounts of energy, resulting in denser ionization tracks and more biological damage [18], not all particles with high LET have equivalent effects. For instance, protons exhibit relatively lower LET values.

RBE is a unitless factor comparing the biological impact of a specific ionizing radiation to a reference, usually X-rays or gamma rays. RBE values vary and are determined through comparative dosing between the test and reference radiation needed to reach a specific biological end-point, such as DNA damage or tumor control [19]. While high LET generally correlates with high RBE due to denser ionization tracks and higher energy deposition [20], this is not uniform across all particle types. For example, protons generally have a consensual RBE of around 1.1, contrasting starkly with the higher RBE values often seen with carbon ions.

Therefore, it is important to recognize that high LET radiation types can vary significantly in their biological impacts, often represented by their RBE values. Such distinctions matter when considering treatment modalities, as higher LET and RBE values such as those of carbon ions can lead to better tumor control and minimized risk of late side effects, compared to protons with lower LET and RBE [17].

## 3. Main Indications of Radiation Therapy

There are two main categories of indications for RT: curative treatments and palliative treatments. The issues regarding the planning, the number of sessions and the expected results are not the same at all.

### 3.1. Irradiation with Curative Intent

Irradiation with curative intent concerns localized or locally advanced cancers. These indications can be divided into three subcategories. Exclusive RT, postoperative RT and pre-operative RT.

#### 3.1.1. Exclusive Radiation Therapy

Exclusive RT can be associated with a concomitant systemic treatment such as chemotherapy, it is called radio-chemotherapy. It is important to understand that in these cases, the tumor was not resected, the doses of RT must be therefore relatively high and the treatments long: up to 41 sessions of treatment for a sacral chordoma for example [21]. Actually, this treatment is proposed either for unresectable localized tumors, as for nasopharyngeal cancers which are surrounded by critical organs and for which the risks of post-operative complications are very high [22], or when the patient has too many co-morbidities and is not operable (the so called “resectable but inoperable” patients), as is the case for some lung cancer patients [23]. Additionally, organ/function sparing serves as a major indication for primary (exclusive) radiation therapy, notably in cases such as laryngeal carcinoma, HPV+ oropharyngeal carcinoma, anal carcinoma, and cervical cancer.

#### 3.1.2. Post-Operative Radiation Therapy

In the post-surgical scenario, where the tumor is macroscopically absent, RT may be employed as a prophylactic measure if the risk of recurrence is high. In cases where a second operative resection is not feasible due to close surgical margins (R2/R1/R0), adjuvant radiochemotherapy is also often employed. These preventative irradiations tend to involve lower doses and shorter treatments compared to exclusive irradiation. For instance, after breast-conserving surgery for breast cancer, a typical regimen would be 50 Gy administered over 25 fractions of 2 Gy each [24]. Similarly, in the context of head and neck cancers with lymph node invasion, the approach would typically involve a dose of 66 Gy distributed over 33 fractions of 2 Gy each [25]. For some cancer locations, targeted therapy can also be used to sensitize tumors to RT, separate from chemotherapy [26]. For instance, in triple-negative breast cancer, the use of PARP inhibitors in combination with adjuvant RT has shown promising results [27].

#### 3.1.3. Pre-Operative Radiation Therapy

Lastly, still in the category of curative irradiation, there are indications of pre-operative RT. In this case, RT is intended to prepare for surgery. The tumor is macroscopically present, but since the patient will be operated on, the doses are lower than in the case of exclusive irradiation. This is the case, for example, for sarcoma [28], esophageal [29], or rectal cancer [30], for which 50 Gy is delivered in 25 fractions of 2 Gy, which is far from the 41 sessions described above.

### 3.2. Ablative Treatments: Stereotactic Options

Ablative treatments such as SRS, SRT, and SBRT represent an emerging category in RT. These are usually considered for primary tumors in patients unsuitable for anesthesia and for specialized cases such as oligometastatic, oligorecurrent, or oligoprogressive diseases. Stereotactic options enable high-precision targeting, which can be critical for tumors located near organs at risk (OARs).

### 3.3. Irradiation with Palliative Intent

The second category of indications refers to palliative indications. In this case, the patients are most frequently metastatic and the objective of the treatment is to treat a symptom. The doses are often much lower, typically 30 Gy in 10 fractions or 20 Gy in 5 fractions [31]. Examples are irradiation of bone metastases for analgesic purposes [32], whole brain irradiation when patients have brain metastases and a risk of intracranial hypertension [33,34], mediastinal irradiation when patients have compressive mediastinal tumors [35], and irradiation for hemostatic purposes when patients have very hemorrhagic tumors [36]. Most of these indications are not urgent. Among the few emergencies in RT, which require treatment as soon as possible, spinal cord compression, superior vena cava syndrome [37], and intracranial hypertension should be kept in mind [38].

### 3.4. Personalized Indications in Radiation Therapy: Balancing Risk and Life Expectancy

In the context of RT for conditions such as HNC, the decision to proceed with treatment should integrate various considerations, including the potential for toxicity. Moreover, specific attention must be given to the patient’s comorbidities and life expectancy. The objective is to achieve personalized treatment planning that draws upon a multidisciplinary approach. This approach aims not only for effective cancer control but also for the optimization of the patient’s quality of life.

## 4. Treatment Planning

Once the decision to treat has been made, treatment planning can begin. The different steps of the treatment if not well explained can appear very confusing to a non-oncology physician. We provide a detailed description of each of them in this section.

### 4.1. Step 1: Simulation CT

The first step of the treatment planning is the simulation CT. This is an essential step. The patients are imaged in the position in which they will be treated, most of the time in supine position. Sometimes, for breast cancer for instance, the patient is placed in lateral [39] or prone decubitus [40]. The challenge will then be to find the same position at each treatment session. Traditional methods often employ tattoos or other skin markers for this purpose. However, emerging technologies such as surface guided radiation therapy (SGRT) are becoming more prevalent, serving as the next step in technical evolution and potentially making traditional markers obsolete [41]. In addition, for several locations such as HNC or brain tumors, restraints are used to ensure that the patient will be immobile during the session [42].

For tumor sites affected by respiratory movements, such as lung, breast, and liver tumors, several techniques have been developed over the last 20 years to consider the different positions of tumors during the respiratory cycle [43]. The first technique is to perform several scans at different times of the respiratory cycle, called 4D-CT, which allows mapping of the tumor at different times of the respiratory cycle. It is then possible to consider these different positions when defining the target volume (see next paragraph). It is also possible to use this 4D-CT to irradiate the tumor only at specific positions, which is called “respiratory gating”. Another method is to teach the patient to stop breathing and only treat at a certain time in the respiratory cycle; this is called breath hold. This technique of deep inspiration breath hold (DIBH) is especially used for left side breast cancers [44] or Hodgkin lymphoma [44]. This allows for a decrease in the dose received by the heart and the lungs. Some modern techniques also make it possible to follow the tumor in real-time during the treatment; this is called tracking.

While simulation CT is the standard for treatment planning in RT, emerging technologies such as simulation MRI and adaptive radiation therapy (ART) are gaining traction. ART allows for daily adjustments based on CT or MRI, offering a more dynamic approach to treatment that is not yet in routine use but represents a promising future direction.

### 4.2. Step 2: Target Volumes and Organ at Risk Delineation

**Figure 2:**
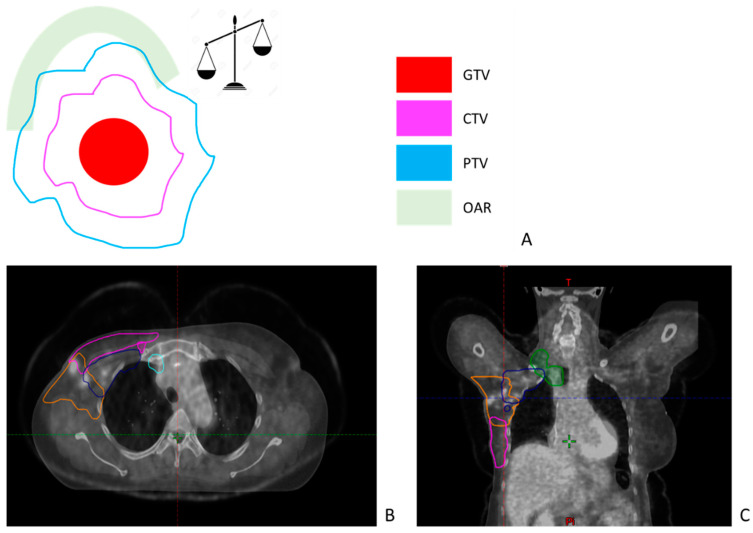
Definition of target volumes and organ at risk. (**A**) The upper part of this figure depicts the process of creating the clinical target volume (CTV) as an anatomical expansion of the gross tumor volume (GTV), and the planning target volume (PTV) as an automatic expansion around the CTV. The lower part of the figure provides the axial (**B**) and coronal (**C**) views of image fusion between the simulation CT and a pre-treatment PET scan for a patient undergoing treatment for breast cancer invading the axillary (purple line), subclavicular (orange and blue lines) and supraclavicular (green line) lymph node regions. The figure emphasizes that the CTV, delineated post-neoadjuvant chemotherapy, and surgery need to encompass all the disease initially visible on the PET scan.

The second step, based on the simulation CT, is the definition of target volumes and organs at risk (Figure 2). This is a crucial step because the entire treatment will be based on the target volume as defined at this stage, which remains the greatest source of uncertainty in RT [45].

Several subsequent reports of the International Commission on Radiation Units & Measurements (ICRU) have defined how these volumes should be defined on the simulation CT [46,47,48]. The most obvious volume is the gross tumor volume (GTV), which is the visible or palpable volume, either in the patient or with the help of imaging (CT, MRI, PET). This volume corresponds strictly to what is perceived, without adding any margin for possible extension. The delineation of the GTV is actually one the main source of uncertainties in RT, with a high interobserver variability (IOV) [45]. Multimodal imaging, including functional and metabolic imaging [49], and artificial intelligence could be useful in this context to decrease the risk of uncertainties and IOV [50,51].

Beyond the GTV, there is often microscopic tumor spread, too small to be seen (even microscopically), but which can be the cause of treatment failure if not addressed. This may be direct dissemination around the tumor or into the draining vessels and lymph nodes. This volume likely to contain microscopic tumor deposits is called the clinical target volume (CTV, Figure 2A) [52]. In the context of a non-resected tumor, the construction of the CTV involves adding a margin around the GTV. This margin is determined based on anatomical considerations, incorporating knowledge of the tumor’s history, and is limited to the boundaries of healthy tissue. In certain tumor locations, such as HNC, a distinction is made between high-risk (HR) and low-risk (LR) CTV. These terms are often used in a clinical context to differentiate between various risk levels within the CTV, but they are not part of the standardized nomenclature suggested by American Association of Physicists in Medicine (AAPM) Report No. 263. The HR-CTV is constructed around the GTV, focusing on the area with the highest risk of recurrence. This region is delineated with precision to ensure that it covers the potential areas of residual disease. The LR-CTV is subsequently constructed around the HR-CTV, encompassing an area with a relatively lower risk of recurrence. This additional volume provides an additional safety margin to account for potential microscopic disease extension beyond the high-risk region. By employing this hierarchical approach in delineating the CTV, the RO aim to effectively target the areas at highest risk while still providing adequate coverage of the surrounding tissues. This approach allows for a more tailored and personalized treatment strategy for patients with non-resected tumors. Emerging deep learning approaches show promise in automating the segmentation of the CTV, aiding ROs in the future. These advanced techniques leverage artificial intelligence and neural networks to accurately delineate the CTV [53,54]. Integration of these tools into clinical practice has the potential to streamline workflow and improve consistency in CTV delineation. In postoperative RT, the GTV was resected by the surgeon. The GTV no longer exists and should therefore (and logically) not be delineated. The CTV is therefore defined as a margin around the tumor bed. In this context, registration between the simulation CT and a pretreatment PET scan may be useful to ensure that the entire initially invaded area is included in the CTV (Figure 2B,C). Numerous guidelines have been developed over the past two decades to assist radiation oncologists in defining the GTV and CTV, aiming to mitigate the risk of previously mentioned uncertainties.

When all these volumes have been delineated, it is possible to select appropriate beam sizes and shapes to adequately cover the tumor (GTV) and microscopic spread (CTV). However, there is an additional source of uncertainty: the reproducibility of patient positioning and the mechanical accuracy of the equipment, the so-called setup errors [55]. It is therefore recommended to add an isotropic margin around the CTV to delineate the provisional target volume (PTV, Figure 2A). The PTV depends on the treatment technique, the device, the location: the margin will be larger if the risk of setup errors is greater. For mobile tumors, as seen previously, several techniques exist to consider the different positions of the tumor during the respiratory cycle. The target volume is sometimes modified to take into account these different positions [56].

Another important step is the delineation of healthy tissues, which in RT are called organs at risk (OAR). The new irradiation techniques allow better protection of these organs. However, these organs must be defined, otherwise the planning software ignores them. Atlases have been validated for the delineation of the organs including for the head, neck, brain, and pelvis [57,58,59]. Moreover, AI-based automatic delineation for OARs is an accepted and broad-used technique, while the already mentioned AI-based automatic delineations of CTV and GTV are in the developmental phase.

### 4.3. Step 3: Definition and Validation of the Radiation Therapy Plan

Once the target volumes and OARs have been delineated on the simulation CT, the patient file is transferred to the physics team. The radiation dosimetrists and medical physicists work to establish the dosimetry with the goal of accurately irradiating the target volume while protecting the OARs as much as possible. Several different techniques are available to achieve this goal. Most RT centers have at least one linear accelerator of electrons (LINAC). These devices can produce photons and electrons with sufficient energy to induce the desired tumor cell and tissue damage. The development of computer technology in particular has led to a considerable evolution of irradiation techniques over the last 20 years, especially for irradiation using high-energy photons, with the development of intensity-modulated radiotherapy (IMRT).

#### 4.3.1. Selection of the RT Technique

Before the development of IMRT, the most widely used radiation technique was three-dimensional conformal radiotherapy (3D-CRT). To design a treatment plan using this technique, the radiation dosimetrist determines the number of beams, the angulation of the beams, and their energy. This technique is still widely used to perform irradiation of simple volumes such as breast cancer without adenopathy or for palliative purposes. Treatment plans using this technique are simple and can be prepared quickly and are therefore useful when there is an indication for emergency RT (metastatic spinal cord compression or whole brain irradiation in case of intracranial hypertension). However, this technique does not allow adequate protection of organs at risk when they are very close to the target, as is the case for parotid glands and brainstem, for patients with HNC.

In IMRT, the planning is called inverse. The planning software automatically determines the number of beams, which can be unlimited (VMAT), and the energy of the beams. The objective is that 95% of the target volume receives at least 95% of the prescribed dose, while respecting the dose constraints to organs at risk, for instance less than a mean dose of 20 Gy in the parotid. Indeed, dosimetric comparison showed that IMRT allowed a better protection of the parotid glands than 3D-CRT [60] and in 2011, Nutting et al. demonstrated that IMRT reduced the risk of xerostomia compared to 3D-CRT [61]. Therefore, IMRT is now currently used for most of the treatment at curative intent, especially for HNC.

Proton therapy offers distinct advantages over IMRT, particularly for target volumes near OARs [Figure 1A,D]. This modality provides a lower dose-bath, aiding in the preservation of normal tissues and reducing radiation-induced secondary malignancies—crucial considerations in pediatric and young adult cases. In HNC, proton therapy has been proven to excel in sparing critical structures such as the parotid gland, mandible, and pharyngeal constrictor muscles [62]. A comparative study showed that intensity-modulated proton therapy (IMPT) led to fewer adverse outcomes, such as feeding tube dependence and severe weight loss, without compromising overall and progression-free survival [63]. However, it is worth noting that proton therapy is not universally superior to IMRT. Due to factors such as robust planning and spread-out Bragg peak (SOBP), the penumbra in proton beams may not always be narrower than in stereotactic photon irradiation. Thus, IMPT might not always offer a dosimetric advantage over photon-based therapies. Choosing between IMPT and IMRT for HNC, for instance, could benefit from models incorporating normal tissue complication probability (NTCP), as proposed by Langendijk et al. [64], alongside traditional dosimetric plan comparisons. Despite these advantages, accessibility to proton therapy remains a challenge. As of 2023, there are only 41 centers in the U.S. and 89 worldwide [65], with priority given to pediatric cases and skull base tumors [66].

#### 4.3.2. Selection of the Fractionation

As previously mentioned, for most of the treatments, 2 Gy are delivered per fraction for 10 to 35 sessions. Over the past several decades, several other fractionations schedules have been proposed. Based on radiobiological principles, for some indications, hyperfractionation with a reduced dose per fraction (1.1–1.5 Gy), administered twice daily, could improve sublethal damage repair in late-reacting tissues and thus reduce radiation-induced late toxicity [67]. Several studies have actually shown that this fractionation could be useful for lung cancer [68] and HNC, especially in the context of a second course of irradiation [69]. Nevertheless, this fractionation remains difficult to implement because it implies that the patient is irradiated twice during the day (once in the morning and once in the evening), which is not always simple from a practical point of view, particularly for patients who live far from the treatment center and cannot be hospitalized. On the other hand, several studies have shown that hypofractionation with a higher dose per fraction (>2 Gy) scheme was possible. This is notably the case for breast cancer treatment. The START trials showed that three weeks of treatment did not induce more late toxicity than five weeks [70] of treatment and more recently, for selected cases, the FAST FORWARD trial showed that one week of treatment did not induce more late side effects without compromising the overall survival [71].

For some techniques, hypofractionation is the standard. This is the case for brachytherapy and stereotactic RT, for which the number of fractions is very limited (one to five fractions) and the dose per fraction very high (up to 20 Gy per fraction for brain metastases treated by radiosurgery). Brachytherapy is probably the oldest RT technique. The principle consists of the insertion of radioactive material in or near the tumor. It is the best technique to ensure the protection of organs at risk. However, it is invasive and usually requires an overnight hospital stay. This technique is often used for prostate or cervical cancer [72]. This technique requires, even more than the others, a long learning curve. An alternative, at least for prostate cancer, is the recent emergence of body stereotactic radiotherapy. This technique allows, for a small target volume, a short treatment (five fractions) with a very high dose per fraction and without increasing the risk of toxicity [73].

Additionally, there are emerging techniques that have the potential to further enhance the previously mentioned risk–benefit balance. One such technique is FLASH irradiation, which has demonstrated promising outcomes in preclinical studies by reducing the risk of lung fibrosis, brain toxicity, and gut toxicity while maintaining the therapeutic benefits for tumor control [74]. Clinical trials are currently being planned to evaluate the efficacy and safety of FLASH irradiation in human patients.

When the dosimetry is completed, the medical physicist must perform a large number of verifications before proposing the dosimetry to the physician. During this initial plan/chart review, the medical physicist should ensure that the prescription follows these guidelines and ensure the fractional dose and total dose in the prescription agrees with the treatment plan [75].

### 4.4. Step 4: Medical Validation of the Treatment Plan

When all these verifications have been completed, the treatment plan is proposed to the physician for last validation before the onset of the treatment. The RO must verify that at least 95% of the target volume receives at least 95% of the prescribed a recommended dose (target volume coverage) and at the same time the dose constraints for OAR sparing are respected. These dose constraints were empirically defined [76] and summarized in the Quantitative Analysis of Normal Tissue Effects in the Clinic (QUANTEC) reports [77]. These dose constraints depend in particular on the type of OAR involved. Classically, a distinction is made between organs at risk in series, for which a maximum dose in a very limited volume can induce a loss of function (such as the spinal cord) and organs at risk in parallel, for which a volume receiving a certain dose (often called VXGy, which means a volume of such organ receiving X Gy) can induce a loss of function (such as the lung). The objective of these dose constraints is to prevent the risk of late toxicity, which classically appears from three months after the end of treatment and disappears only very slowly or never.

**Figure 3:**
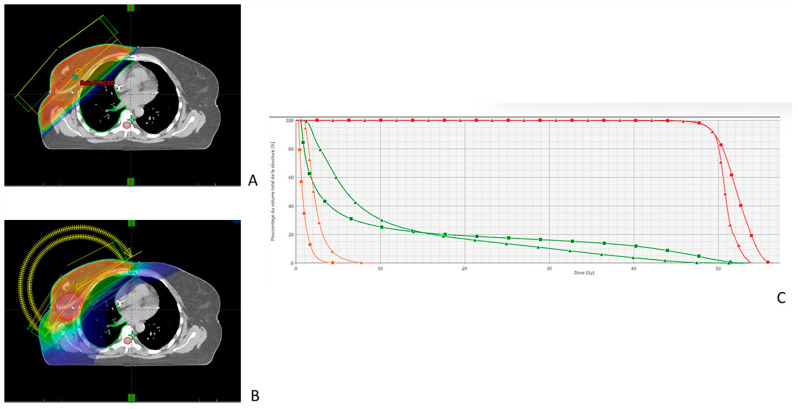
Dosimetric Comparison of 3D-CRT and IMRT (VMAT) for Right Breast Cancer Treatment. The figure showcases a comparative evaluation of the 3D conformal radiotherapy (3D-CRT) (**A**) and intensity-modulated radiation therapy (VMAT) (**B**) plans for treating right breast cancer. In the axial views on the left, high dose areas are represented by warm colors and low dose areas by cold colors. The IMRT plan appears to decrease the high dose area in the right lung but increases the low dose in the right lung, heart, and contralateral breast. The dose-volume histogram (**C**) confirms this observation. The red lines symbolize target volumes (squares for 3D-CRT and triangles for IMRT), indicating similar coverage with both techniques. For the heart (orange lines), the dose is lower with the 3D-CRT plan. As for the right lung (green lines), the lung volume receiving a low dose is larger in the IMRT plan, while the volume receiving a high dose (V20 Gy, V30 Gy) is larger in the 3D-CRT plan.

**Figure 4:**
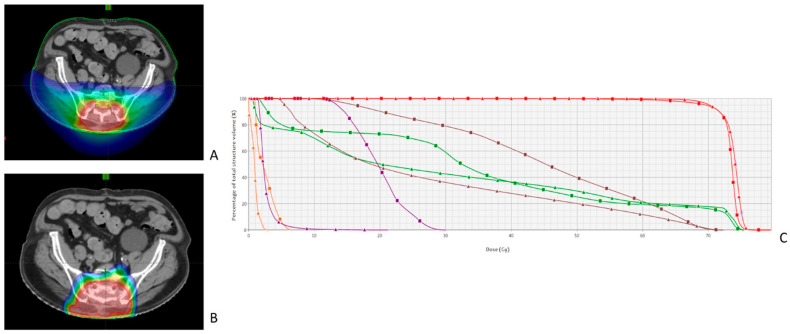
Dosimetric Comparison between IMRT (Tomotherapy) and Proton Therapy for Sacral Chordoma Treatment. This figure illustrates a comparative assessment of the intensity-modulated radiation therapy (Tomotherapy) (**A**) and proton therapy (**B**) plans for treating a sacral chordoma. In the axial views on the left, high dose areas are represented by warm colors and low dose areas by cold colors. The proton therapy plan seems to decrease the dose received by muscles, bones, and abdominal cavity, but increases the dose in skin and subcutaneous tissues. The dose-volume histogram (**C**) indicates that both techniques (triangles for proton and squares for IMRT) adequately cover the target volumes (red lines). For the rectum (brown lines), bladder (purple lines), and intestine (green lines), doses are lower with proton therapy than with photon therapy.

For this step, the RO usefully uses a diagram called a dose-volume histogram (DVH) with the volume of an organ on the ordinate and the dose received by that organ on the abscissa. At this point, the RO often has to choose between different techniques, as illustrated in Figure 3, which compared 3D-CRT and VMAT for the treatment of breast cancer, and Figure 4, which compared VMAT and proton therapy for the treatment of a sacral chordoma. For some cases, the choice is quite easy [78], but in some cases the choice is more difficult. Consider the case of a patient with a tumor completely in contact with the parotid gland; the choice will be between irradiating the target volume completely with the risk of inducing severe late oral dryness or better protecting the parotid gland with a higher risk of local recurrence.

## 5. Treatment Delivery and Follow-Up

When the best plan is obtained considering the benefit/risk balance, the treatment can begin.

### 5.1. Image-Guided Radiation Therapy

During the treatment course, several images are used to ensure that the patient is well positioned and to reduce the risk of setup errors. This is called image-guided radiation therapy (IGRT). However, the longer the patient remains on the treatment table during image capture, the higher the potential for setup errors due to patient motion. Therefore, it is essential to minimize the time elapsed between image capture and treatment decision-making. Incorporating deep learning algorithms can be beneficial for adjusting for patient motion during this process [79].

The simplest images, used for the past 50 years, are 2D radiographs with at least two orthogonal incidences. These radiographs are compared with radiographs obtained from the simulation CT (called DRR) to calculate the displacements of the patient and make necessary modifications so that the treatment is delivered as accurately as planned. In 2010, Van Beek showed that automatic alignment between the two types of images was possible and could reduce this delay between image acquisition and processing [80]. The devices also provide an image of the treatment beam which allows a final check before starting the treatment. Furthermore, as previously mentioned, surface guided radiation therapy (SGRT) utilizes real-time surface imaging to refine patient positioning, which aligns with the overarching goal of enhancing the accuracy of radiation delivery.

Another type of image often used to decrease the risk of setup errors is cone beam CT (CBCT). The CBCT obtained on the treatment table can be easily compared to the simulation CT [81]. This type of image is also useful to follow certain organs during treatment and provide treatment adaptation if necessary [82]. Indeed, patients treated for HNC have a high risk of weight loss during treatment. IGRT may be useful to compare the position of the parotid glands on the simulation scan and on this CBCT performed after one month of treatment. If the position of parotids has completely changed, there is a high risk of overdosing and then xerostomia. In this case, it is common to provide a new treatment plan adapted to the new patient morphology.

Finally, MRI guidance in radiation therapy (MRgRT) marks a significant advancement in treatment precision, notably enabling daily adapted radiation therapy (ART). Its main applications are seen in head and neck cancer (HNC) [83,84,85] and prostate adenocarcinoma [86]. One of the major advantages of MRgRT is its capability to minimize dose to OARs through daily treatment adaptations. Although ART is resource- and time-intensive, the substantial reduction in OAR dose highlights its value [87]. This method is particularly beneficial for patients undergoing reirradiation, allowing for decreased dose to OARs [88]. Overall, MRgRT enhances the quality of care, thereby reducing adverse effects in patients undergoing RT.

### 5.2. The Role of Radiation Therapists in Treatment Delivery

Radiation therapists (RTTs) are an integral part of the radiation oncology team, bridging the gap between treatment planning and delivery. Tasked with implementing the radiation treatment plan prescribed by the radiation oncologist, RTTs ensure accurate patient setup and precise radiation dose delivery. They are skilled in the operation of complex radiation equipment and are trained to make real-time adjustments for anatomical variations, thereby safeguarding both treatment efficacy and patient safety. Their expertise contributes significantly to the overall success of radiation therapy protocols.

### 5.3. Management of Acute Toxicity

**Figure 5:**
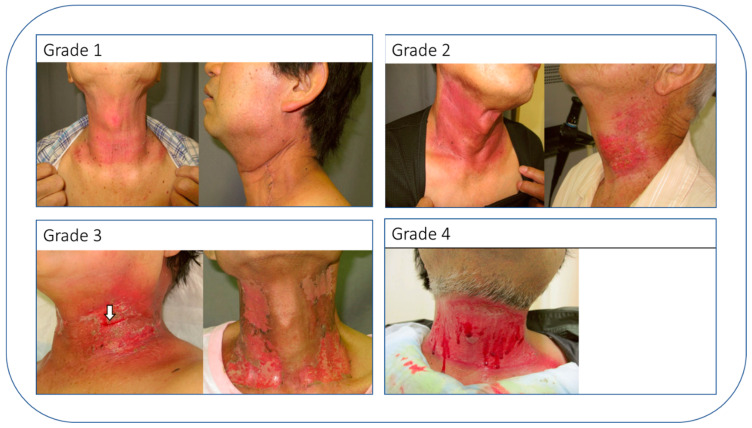
Classification of Radiation Dermatitis Post Head and Neck Cancer Radiation Therapy. Adapted from Zenda et al. [90] (PMID: 26850926), this figure displays the four grades of radiation dermatitis that can develop following radiation therapy for head and neck cancer. Grade 4, which presents the highest severity, carries a substantial risk of infection necessitating treatment interruption for multiple days.

During the treatment phase, it falls on the radiation oncologist (RO) to closely track and manage acute side effects that may arise. These acute toxicities generally appear during the RT course and are usually resolved within a 3-month period post-treatment. Often resulting from the death of fast-dividing cells such as those in the skin or mucous membranes, these toxicities show a dose-dependent relationship. While various grading systems exist, leading to some inconsistencies in retrospective studies [89], the Common Terminology Criteria for Adverse Events v. 5 (CTCAEv5) is most commonly employed (https://ctep.cancer.gov/protocolDevelopment/electronic_applications/ctc.htm, accessed on 30 October 2020). Prompt intervention is key to minimizing the risk of serious complications. For example, in HNC cases, a late-stage grade 2 skin toxicity might be considered manageable, whereas grade 4 (life-threatening) or grade 5 (fatal) toxicities at any point are definitively not acceptable (Figure 5). This is due to the fact that grade 4 toxicity not only causes significant pain and discomfort for the patient, but also necessitates treatment interruption, which in turn increases the risk of tumor repopulation and diminishes treatment efficacy [90,91]. At the completion of RT, the RO should prepare a treatment (completion) summary [92]. This report details the indication and technical characteristics of the treatment, specifying the acute toxicities that occurred during the treatment and the doses received by the organs at risk likely to induce late toxicities. This report should be sent to the patient’s primary care physician and to all non-oncology colleagues involved in the diagnosis of cancer, to initiate long-term patient follow-up. This report is an essential document, and it seems important that all physicians for whom it is intended understand it and will be able to use it.

### 5.4. Follow-Up in Radiation Oncology

**Figure 6:**
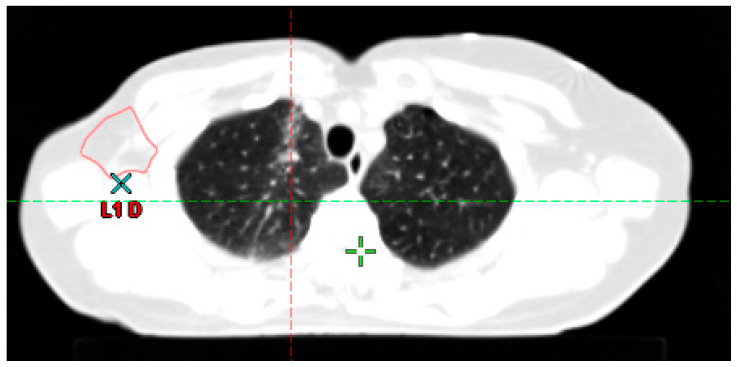
Radiation-Induced Fibrosis Post Right Breast Cancer Radiation Therapy. This figure presents a case of a patient who underwent radiation therapy for right breast cancer, with the subclavicular lymph node area included in the target volume. A lung CT scan, conducted six months post-treatment, shows an opacity in the right upper lobe indicative of radiation-induced fibrosis. It is crucial to distinguish this from potential lung infection.

After completion of treatment, patients should be followed by the radiation oncologist in collaboration with the medical oncologist and the patient’s primary care physician. The objectives of this follow-up are: (1) To detect locoregional recurrence as early as possible, (2) to detect and manage treatment-related complications, (3) to motivate patients to continue adjuvant treatment (such as hormone therapy for breast cancer), and (4) to provide psychological support and information to help patients return to a normal life after cancer [93]. Figure 6 illustrates the development of late lung fibrosis six months after the completion of the RT in a patient treated for breast cancer including sub clavicular lymph nodes. This image can explain some symptoms as dyspnea or chest pain.

## 6. Conclusions

Numerous studies have demonstrated the pivotal role of multidisciplinary management, including the importance of an interdisciplinary team (IDT), in enhancing cancer diagnosis and treatment across all sites [94]. This approach typically involves a team of physicians, including medical oncologists, surgeons, primary care physicians, and radiation oncologists (ROs), who possess a comprehensive understanding of radiation oncology practices. The IDT additionally brings in medical physicists, radiologists, and other healthcare professionals to ensure a well-rounded approach to patient care. Multidisciplinary management also encompasses non-oncologists who engage with patients at various stages of their treatment journey. These professionals may not be as familiar with RT, leading to potential confusion surrounding the technical information presented in end-of-treatment reports. Recognizing the need for improved collaboration and understanding, it is essential to consider the transformative potential of advancements in imaging and artificial intelligence (AI) for radiation oncology planning and delivery. The integration of these cutting-edge technologies can pave the way for more precise, personalized, and efficient treatments, ultimately fostering better teamwork among multidisciplinary teams, including IDTs. To support this goal, the present study serves as a valuable resource for non-oncologists, medical students, and researchers involved in cancer treatment, offering insight into the intricacies of RT. By providing these professionals with a solid understanding of RT, we can facilitate better collaboration among all medical practitioners responsible for the same patient, ultimately promoting more effective and coordinated care.

## Data Availability

Research data are stored in an institutional repository and will be shared upon request to the corresponding author.
